# Attitudes and perceived barriers to evidence-based practice among occupational therapists in Jordan

**DOI:** 10.1371/journal.pone.0299013

**Published:** 2024-05-23

**Authors:** Dua’a Akram Alwawi, Majd Jarrar, Somaya Malkawi

**Affiliations:** Department of Occupational Therapy, School of Rehabilitation Sciences, The University of Jordan, Amman, Jordan; University of Hafr Al-Batin, SAUDI ARABIA

## Abstract

Evidence-based practice (EBP) refers to the clinical decision-making process incorporating the best available evidence from research, therapists’ clinical experience, and patient values. The current study aimed to examine the experience of Jordanian occupational therapy practitioners (OTs) in using EBP and to identify the perceived barriers to implementing EBP among OTs in Jordan. The study utilized a cross-sectional descriptive study design. A questionnaire was emailed to OTs who have been working in Jordan for the last six months of their practice in a clinical setting. The majority of the participants had a positive attitude toward EBP. However, they reported several barriers to implementing EBP, including a lack of tools and equipment in clinical settings as a major barrier (65.8%). National collaborative actions are needed to develop strategies to improve the utilization of EBP in occupational therapy (OT) practice and to overcome the barriers therapists experience with implementing EBP.

## Introduction

Evidence-based practice (EBP) refers to the clinical decision-making process incorporating the best available evidence in research, therapists’ clinical experience, and patient values [1]. EBP has spread into the healthcare field due to the realization of the importance of using valid and reliable information based on scientific evidence in the diagnosis, therapy, and prognosis of different diseases [2]. EBP aims to improve therapy outcomes and patient quality of life while reducing associated healthcare costs [2,3]. Occupational therapy practitioners (OTs) began using EBP in making clinical decisions to improve the quality and effectiveness of the care they provided to their patients [4]. Therefore, a major push has been made toward using EBP for OTs in routine clinical care [5].

Several studies have investigated the attitude of OTs toward EBP [5–21] These studies indicated that most OTs had a positive attitude toward EBP and agreed on its importance in practice and clinical decision-making. However, therapists’ attitude was not reflected in their behavior using EBP in clinical practice. Therapists reported clinical experience [10,11,22] and the knowledge of their colleagues [15,22,23] as a primary source of information to make related clinical decisions.

Therapists in previous studies identified different factors that challenge research utilization; some of these factors are related to the lack of skills needed to translate research results into practice [15,23–25], while others related to the organizational factors such as departmental support [26], lack of time and lack of access to research articles [6,7,10,13,15]. These studies were carried out in several contexts where the occupational therapy profession has different professional organizations and legislation levels. However, because no comparable research has been conducted in Jordan, there may be limitations to the findings’ generalizability and applicability to Jordanian OTs. Additionally, according to Kristensen et al [27], culture affects the implementation of EBP in healthcare practices, which might be an additional reason to conduct this study.

Occupational therapy (OT) is considered a developing profession in Jordan as it was introduced into education in 1999 [28]. To date, three governmental universities that offer OT programs accredited by the World Federation of Occupational Therapy (WFOT) graduated (1751) occupational therapists. All three universities teach occupational therapy primarily in English [28]. The highest percentage of Jordanian occupational therapists practice in hospitals and pediatric settings [28]. In Jordan, OTs are not able to work as autonomous practitioners and are not allowed to have direct access to patient management without getting a referral from a physician. Moreover, some centers impose policies limiting the OTs’ flexibility in providing the type of services they think are better for the patient [28]. One of the ways to improve the standing and value of the OT profession in Jordan is by incorporating EBP into clinical practice [29]. The occupational therapy profession may benefit from identifying EBP barriers by attracting the attention of work facilities, educational sectors, and other stakeholders [30].

Although previous studies have investigated the satisfaction and current working conditions of Jordanian OTs [28,31], no studies have been completed to focus on OTs’ perceptions toward integrating EBP in clinical settings utilizing a sample of Jordanian OTs. Therefore, the current study aims to examine the experience of Jordanian OTs in using EBP.

Research questions

1. What is the Jordanian OTs’ perception of EBP?
2. Which sources of evidence do Jordanian OTs use in making clinical decisions?
3. What are the barriers Jordanian OTs experience when implementing the EBP?

## Methods

Ethical approval was granted by the Board of the Occupational Therapy Department and the Board of the Rehabilitation Sciences Faculty at the University of Jordan on September 16^th^, 2020 (496/2020/19). We followed the Strengthening the Reporting of Observational studies in Epidemiology (STROBE) checklist (S1 File) for reporting this study.

### Participants

The target population for this study was Jordanian OTs who have worked in clinical practice. To be in the study, a person had to work in Jordan as an OT for the last six months of their practice in a clinical setting. Participants were recruited from private and public institutions.

### Study design and instrumentation

This study utilized a cross-sectional descriptive study design. The authors developed an English-language questionnaire based on the reviewed available literature (S2 File). The survey questions were developed from previously validated questionnaires addressing the EBP among OTs and other healthcare professionals in different [15,32]. The survey was piloted with six OTs from different settings in Jordan to ensure the format of the questions was understandable and appropriate to Jordanian clinical settings. Close-ended questions were used to collect demographic information, perceptions, attitudes toward EBP in clinical settings, and resources therapists use in clinical decision-making. The survey also examined the barriers to EBP utilization in clinical settings. The researchers designed an online survey using Google Forms.

The survey consisted of five sections. The first section focused on demographic information. In the second section, participants were asked to rate how often they used sources of information by responding to a rating scale of "Always, Often, Sometimes, Rarely, Never, and No Access.” The third and fourth sections focused on the participants’ perceptions regarding EBP and barriers that influence utilizing EBP; both sections were measured by responding to multiple statements on a five-point Likert scale (“Strongly Agree,” “Agree,” “Neither agree nor disagree,” “Disagree,” “Strongly disagree”). Based on their opinion, the therapists were also asked to rank the three most significant barriers to integrating EBP in clinical decision-making using a Guttman rank-ordering scale. In the last section, participants were asked to indicate the percentage of clinical decision-making time that involves EBP by responding to multiple-choice answers involving categories of percentages. At the end of the last section, participants were asked to mention strategies that would help them incorporate EBP in their clinical decision-making.

### Data collection and analysis

Convenience and snowball sampling techniques were used to collect data. Data collection started on October 15^th^, 2020, and ended on 15^th^ December 2020. The researchers emailed the survey link to the contact persons from 15 clinical settings listed in the University of Jordan Occupational Therapy Department Fieldwork Database. The clinical settings were from Jordan’s Northern, Central, and Southern areas. Contact people were asked to forward the survey to OTs working at their sites and other sites not listed in the database. Participants received an e-mail that contained information about the study, inviting them to participate in the study. This e-mail explained to participants the goal of the study and the importance of their participation and asked their permission to participate in the study. Participants indicate their consent by entering and completing the survey.

All descriptive analysis was performed using the Statistical Package for Social Sciences (SPSS) version 28 [33]. To simplify the results, some response categories were collapsed. Specifically, in the second section, the response categories were collapsed into: Most of the time, Sometimes, Rarely to Never, and No Access to this source. The third and the fourth sections’ response categories were collapsed into Agree, Neutral, and Disagree.

## Results

Of the 120 surveys sent to OTs, a total of 79 surveys were completed. Table 1 presents the demographic characteristics of the OTs who participated in this study. Most of the participants were females (*n* = 68, 86.1%). While around half of all participants were 18–24 years old (*n* = 38, 48.1%), and one third of all participants had 1–2 years of experience (*n* = 22, 27.8%). The highest percentage of participants reported working in outpatient clinics for adults, 34.2% (*n* = 27). Most participants (n = 50, 63.3%) worked in settings with more than two OTs. Furthermore, most participants (*n* = 66, 83.5%) stated they held a bachelor’s degree in OT, while eight participants (10.1%) reported having a graduate-level degree.

**Table 1:** Demographic characteristics of occupational therapists (N = 79).

Variable		*n*
Age		
	18–24	38
	25–34	30
	35–44	10
	45–54	1
Gender		
	Male	11
	Female	68
Practice years		
	< 1	19
	1–2	22
	3–4	13
	5–10	13
	11–15	7
	>15	5
Highest degree		
	Three years Diploma	5
	Bachelor	66
	Masters	6
	PhD	2
Work Setting		
	Out-patient clinic adults	27
	Hospital	17
	School	8
	Community based center	1
	Mental health	3
	Out-patient clinic pediatrics	16
	Freelance	7
Number of OTs in the setting		
	1	14
	2	15
	>2	50
Number of working hours		
	< 20	28
	20–39	30
	> 39	21
Affiliated with a professional association		
	Yes	8
	No	71

Note: OTs: Occupational Therapists.

Most participants worked in the capital of Jordan, Amman *(n* = 69,87.4%). The rest of the participants worked in other major cities of Jordan, while none of the respondents worked in the South of Jordan.

### Sources used by OTs in clinical decision-making

Table 2 displays the sources of information OTs who participated in this study used in clinical decision-making. The majority of respondents (72.2%) reported that they used clinical experience as a significant source of information for clinical decision-making, followed by utilizing textbooks (45.6%) and information obtained from conferences and workshops (45.6%). Most respondents reported that they rarely or never used information obtained from non-occupational therapy colleagues (60.8%). The relationship between educational level and the source of information was investigated using chi-square, and it was found that it was not significant. Furthermore, the relationship between the source of information and the setting was not significant.

**Table 2:** Sources of information used by OTs* in clinical decision making (N = 79).

Source of Information	Most of the time (%)	Sometimes (%)	Rarely to never (%)	No access to this source (%)
Information obtained from clinical experience	72.2	24.1	1.3	2.5
Information obtained from conferences and workshops	45.6	29.1	24.1	1.3
Information obtained from reviewing research articles	38	24.1	35.4	2.5
Information obtained from textbooks	45.6	36.7	17.7	0.0
Information obtained from occupational therapy colleagues	40.5	34.2	24.1	1.3
Information obtained from non-occupational therapy colleagues	7.6	29.1	60.8	2.5
Information obtained from web resources	36.7	44.3	19.0	0.0

Note

*OTs Occupational Therapists.

### Perceptions and attitudes towards EBP

The perceptions and attitudes of OTs toward EBP are shown in Table 3. The vast majority of the participants believed that EBP helped with clinical decision-making (91.1%), it could play a positive role in clinical practice (88.6%), and it improved patient outcomes (87.3%). Only 8.9% of participants believed that EBP is client-centered.

**Table 3:** OTs’ attitudes and perceptions toward EBP (N = 79).

Perceptions and attitudes towards EBP*	Agree (%)	Neutral (%)	Disagree (%)
EBP can play a positive role in clinical practice	88.6	11.4	0.0
EBP helps with clinical decision-making	91.1	8.9	0.0
EBP improves patient outcomes	87.3	12.7	0.0
I would like to use evidence in clinical practice.	82.3	16.5	1.3
Academics are more likely to use EBP than clinicians	64.6	26.6	8.9
EBP is difficult to use in clinical practice	25.3	32.9	41.8
The profession should emphasize research in its education	81.0	17.7	1.3
EBP devalues practitioners’ clinical experience	29.1	27.8	43.0
EBP removes creativity from practice.	24.1	26.6	49.4
Research is essential for demonstrating the efficacy of interventions	74.7	21.5	3.8
Research and clinical experience are equally important	82.3	13.9	3.8
EBP is client centered	8.9	41.8	48.1

Note

*EBP Evidence Based Practice.

### Barriers

Table 4 displays the barriers that influenced Jordanian OTs’ use of EBP in a clinical setting. The results showed that the most frequent barriers to integrating EBP in clinical practice were the inadequate available tools and equipment in clinical setting for implementing EBP (65.8%), the lack of incentive for using EBP (55.7%), the lack of skills for evaluating and understanding the results of research (48.1%), and not having enough authority to implement new ideas from the literature into practice (46.8%). Furthermore, many participants expressed that it is difficult to change their established patterns of practice (45.6%), and there needs to be more time to implement new ideas obtained from the research (44.3%).

**Table 4:** Barriers to incorporating research into practice (N = 79).

Barriers of EBP*	Agree (%)	Neutral (%)	Disagree (%)
Occupational therapists do not have time to read research	36.7	20.3	43.0
There is limited access to research articles.	15.2	27.8	57.0
Occupational therapists lack skills for locating the best research evidence	13.9	30.4	54.4
Occupational therapists lack skills for evaluating and understanding results of research	48.1	21.5	30.4
Occupational therapists do not have enough authority to implement new ideas from the literature into practice	46.8	22.8	30.4
Reimbursement is based on quantity of cases instead of quality of intervention	35.4	44.3	20.3
There is insufficient time to implement new ideas obtained from research	44.3	32.9	22.8
Occupational therapists find it difficult to change their established patterns of practice	45.6	19.0	35.4
Tools and equipment in clinical settings are inadequate for the implementation of EBP	65.8	31.6	2.5
There is a lack of incentive for using evidence-based practice	55.7	35.4	8.9

Note

*EBP Evidence Based Practice.

### Utilizing EBP in clinical decision-making

**Fig 1:**
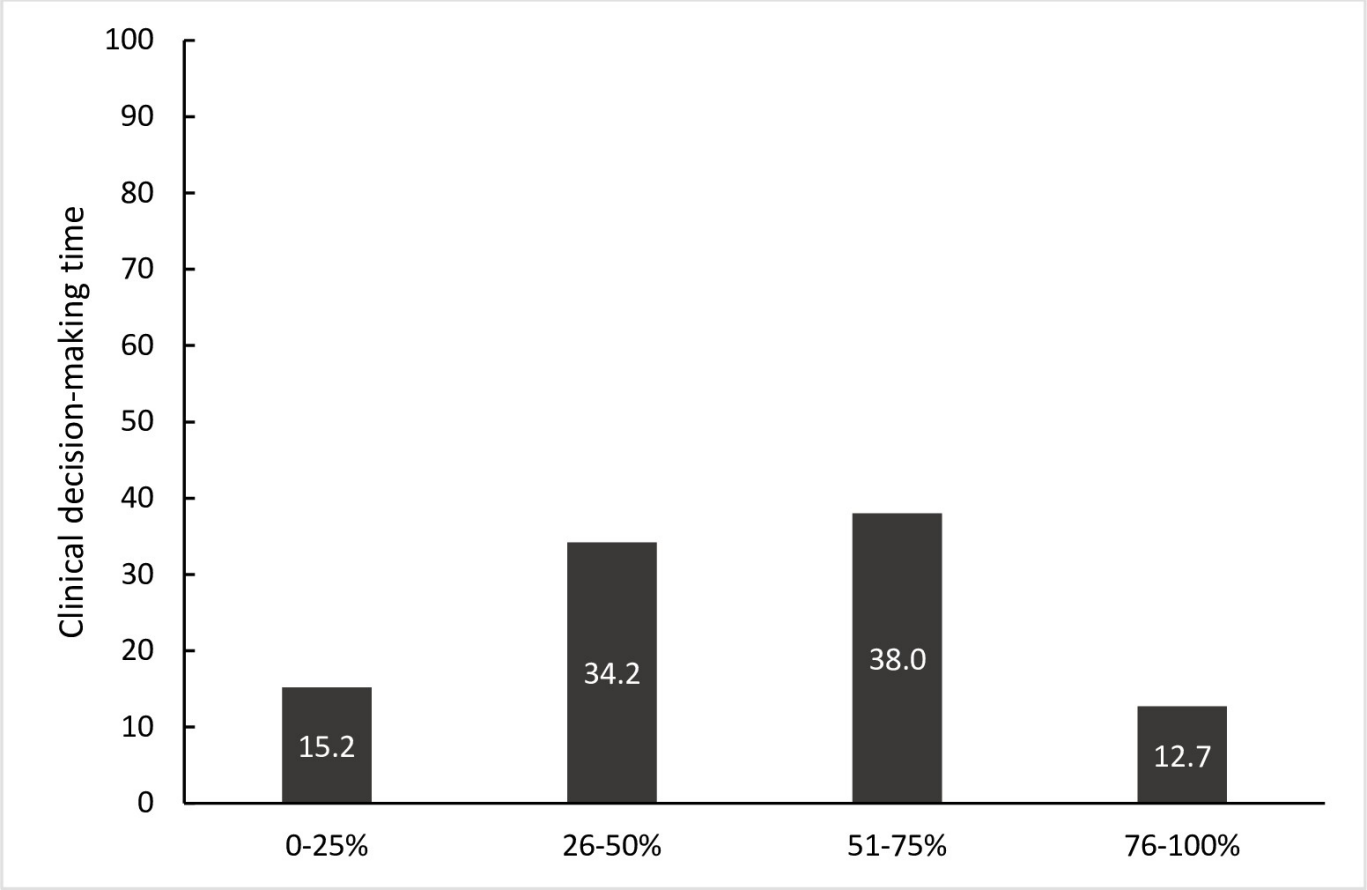
Percentage of clinical decision-making time that involves EBP.

Fig 1 displays the approximate percentage of time participants spent utilizing EBP in clinical practice. Most respondents reported using EBP for 51–75% of their clinical decision-making time. When asked about resources that may help them better incorporate EBP in clinical practice, one respondent stated, “Training and education on incorporating EBP in clinical practice would be a good start to enhance utilizing EBP.” Another respondent said, “If my supervisor in the clinic suggests reading a textbook about EBP, I will be more willing to use EBP in my treatment.” Yet another therapist stated, “If I have access to research articles on OT treatment and assessment process in the clinic, I will definitely use EBP in treatment more often.” Another area highlighted by another participant related to the lack of time to read EBP; the respondent noted, “decreasing the huge amount of my caseload and providing me with more time to read research articles will help me use EBP in clinical practice.” The final area that participants emphasized was the limited authority to use new ideas from EBP since there was a lack of awareness of the role of OTs in rehabilitation settings. One respondent said, “I think increasing the awareness about the role of OTs in the rehabilitation settings would give me the chance and power to use new strategies in the treatment suggested by research articles.” Based on participants’ responses, most reported factors that influenced utilizing EBP related to the environment, such as lack of tools and equipment or factors related to lack of required skills to implement EBP. Table 5 summarizes resources reported by participants to improve incorporating EBP in clinical decision-making.

**Table 5:** Resources would help the therapist to incorporate evidence-based practice.

Access to Journal articles and Journals	9
Books	4
Community centers	1
workshops and networking	5
Internet	8
communication with academic departments	2
workshops that focus on research	2

## Discussion

Occupational therapy is a young profession in Jordan. This study was the first study designed to understand better Jordanian OTs’ attitudes toward EBP and the barriers influencing the integration of evidence-based practice into clinical decision-making.

Most respondents were OTs who held a bachelor’s degree in occupational therapy. This is expected since no post-professional occupational therapy programs are offered at Jordanian universities. The years of experience for respondents reflect the young nature of the occupational therapy profession in Jordan. These results are consistent with previous studies conducted in Jordan involving occupational therapists [31,34].

In line with previous studies, participants prefer to use their clinical experience [10] and textbooks as the most frequent primary source of information to implement EBP. Therapists indicated the importance of using clinical expertise and other sources of information in conjunction with EBP to individualize the treatment provided based on case-by-case evaluation [35,36]. Evidence from research would never replace clinical expertise; instead, it would support decisions based on understanding clients’ needs and priorities [36]. Using clinical experience as the most frequent source of information was surprising especially that most of the study participants had less than two years of experience. This might be an indication that participants are overestimating the extent of their experience.

In contrast to previous studies [14,21]), the participants in this study did not consider non-occupational therapy colleagues as a worthy source of information. This could be justified by insufficient interprofessional education (IPE) teaching across occupational therapy curricula in Jordan. Although the importance of working within interdisciplinary teams is highlighted in some theoretical courses, it is not well articulated and implemented throughout the curriculum. Efforts should be targeted toward integrating IPE into the curriculum and creating opportunities for students to implement IPE in clinical placement. Another reason that could contribute to this result is the restrictions imposed by workplace policies that hinder the collaboration between professionals [30,33].

Many OT models put the client and his needs at the center of the decision-making process. The essence of the client-centered approach is to provide services congruent with the person’s values, interests, and priorities. The literature has shown that EBP and client-centered therapy complement each other for clinical decision-making [37]. However, most respondents reported that EBP does not support client-centered therapy. This result could reflect how EBP has been defined and taught in Jordan. Schools in Jordanian universities that teach occupational therapy need to revise the way they teach EBP in their curricula to reflect that EBP is considering patients’ values and priorities. Then, EBP needs to be integrated into other courses across the curriculum, so the students know how to implement and integrate EBP in clinical decision-making. This aligns with the guidelines outlined in the policy document from the WFOT, advocating for a curriculum that encompasses EBP [38].

Consistent with previous research from other countries regarding utilizing EBP in clinical practice, our results indicated that Jordanian OTs have a positive attitude toward integrating EBP into treatment [5,9,12,14,16]. Despite the positive attitude toward EBP, respondents agreed that implementing EBP is challenging, especially in regard to transferring findings from research into practice. To help overcome this barrier, efforts should be directed toward providing EBP skill training throughout therapists’ careers. Clinical guidelines that provide evidence-informed recommendations, which summarize and appraise research findings, would also help utilize EBP. Moreover, encouraging therapists to write plain language summaries for their research would help translation knowledge.

OTs in this study indicated that they could access, search, and locate the best available evidence. However, they reported a need for more skills in evaluating the quality of research and applying its findings to clinical practice. This is surprising as most of the study participants were from the younger generation who have learned the fundamentals of EBP from the OT curriculum [39]. This might indicate that educators need to emphasize better skills of evaluating, criticizing, and transferring research findings into their curriculum.

A unique barrier to implementing evidence-based practice in Jordan is the lack of tools and equipment. OT clinics are not equipped with the tools and equipment required to implement research findings in Jordan and other middle- and low-income countries. Jordanian therapists reported a lack of resources available at the OT clinics regarding assessment tools, therapy tools, and equipment (e.g., splinting materials, and heat guns) [40]. Resources needed for providing quality care in Jordan are limited regardless of the setting (e.g., private centers or public hospitals). The shortage of these items could lead therapists to use traditional therapy that evidence may not support. National and international efforts should be channeled to enrich rehabilitation services by providing funding to meet the needs of therapists to implement EBP.

Moreover, cultural aspects among patients and healthcare providers would impact the OT practice in Jordan and the ability to utilize EBP [40,41] The common culture in Jordan supports the medical model of occupation-based activities (OBA), limiting the adoption of the recommended interventions that support OBA. Malkawi et al [40] argued that patients prefer exercising and taking medications over using OBA. When transferring, research findings that support OBA, patients may reject it, leading therapists to favor traditional treatment. Starting with the academic curricula of all healthcare professions, efforts should be directed to raise awareness about the role of OTs among the public and healthcare team members to help OTs align with their identity [42].

Another significant barrier reported was the lack of organizational support and encouragement to utilize EBP. A similar pattern of results was obtained from another profession where physiotherapists from the United Arab Emirates (UAE) also reported the lack of support and encouragement as a major barrier to EBP [43]. Internationally, the organizational system does not provide incentives for a better quality of care nor encourages interventions based on supporting evidence to motivate therapists to use the most updated research evidence [38,43,44]. Furthermore, the organizational system in Jordan minimizes the opportunities for discussion among medical team members, often resulting in imposing the physician’s orders. This could be an additional barrier to implement EBP among OTs.

The lack of affiliation with professional associations could be an additional barrier to utilizing evidence in practice. Those who are not affiliated with professional associations could feel isolated from the OT community as they are less likely to be aware of continuing education opportunities and professional events. Although we did not explore the association between professional affiliation and perception of EBP due to the small sample size, this factor needs to be explored in future studies.

EBP implementation is multifaceted: confidence, tools, time, etc. There are no available research studies on Jordanian OTs’ skills in utilizing EBP. This study provided the first step toward better understanding the barriers to implementing EBP. Future studies should examine strategies to improve the applicability of research findings in clinical settings, as most of the study participants reported difficulty implementing research findings in clinical settings.

This study provides valuable information about the attitudes of Jordanian OTs toward utilizing EBP, but it has some limitations. First, the small sample size was one of the significant limitations of this study. Second, all respondents were from Jordan’s central and northern cities, and no responses were received from southern cities. This would be explained by the lack of rehabilitation centers that employed OTs in the southern cities; therefore, the study results may not be generalized to therapists in the southern cities of Jordan. Consequently, future studies need to investigate their attitudes toward EBP and the barriers they experience to implementing EBP. They may have different experiences than therapists from Jordan’s Central and Northern areas.

Implementing EBP requires a multilayered strategy that includes actions at several levels: education, practice, management, and policy-making. We recommend that for EBP to work in Jordan, the curriculums of undergraduate and post-graduate programs should be refined and modified to have more EBP content in both theoretical and practical training. Moreover, collaborations between educators and therapists should be facilitated, such as holding journal clubs, regular research meetings, and continuing education opportunities.

Furthermore, immediate organizational and infrastructure solutions should be integrated into practice areas that promote EBP; this includes providing computer and internet access to medical libraries and allocating budget and funding resources to equip OT clinics with needed tools and materials. Finally, enhancing the supportive practice culture that embraces EBP at the managerial level is required to facilitate EBP.

## Conclusion

This study indicated that Jordanian OTs have a positive attitude toward utilizing EBP in their treatment; however, they reported several barriers that influence the integration of EBP into clinical practice. The most significant reported barriers were the lack of tools and equipment in the clinical setting and organizational support to implement EBP. National collaborative actions are needed to develop strategies to improve the utilization of EBP in OT practice and overcome the barriers therapists experience to implementing EBP.

## Supporting information

S1 File STROBE checklist for the EBP study. (DOCX)

S2 File Evidence based practice survey. (DOCX)
