# Supplementary material for: Attitudes and perceived barriers to evidence-based practice among occupational therapists in Jordan
Source: PLoS One. 2024 May 23;19(5):e0299013. doi: 10.1371/journal.pone.0299013 (PMC11115225; doi:10.1371/journal.pone.0299013)
Supplement: S2 File — (DOCX) [file pone.0299013.s002.docx]

**The University of Jordan**

**Department of Occupational Therapy**

**Study Title**

Jordanian Occupational Therapists’ Perceptions of Utilizing Evidence-Based Practice: A Survey

**Section 1**: Background information

For this section, I would like to know about you and your experience as an Occupational Therapist (OT). For the following questions, please select the response that best describe you.

1. How old are you?

- 18-24
- 25-34
- 35-44
- 45-54
- 55-64
- >65

1. What is your gender?
   - Male
   - Female
2. How many years have you been practicing as an Occupational Therapist (OT)?

- < 1 year
- 1-2 years
- 3-4 years
- 5-10 years
- 11-15
- > 15 years

1. Please indicate your highest degree in the field of Occupational therapy

- Associate’s degree
- B.S
- M.S
- PhD
- Other (please specify)__________________________________

1. In what setting do you work? (please check all that apply)

- Rehabilitation center
- Academic hospital
- Non-academic hospital
- School based center
- Community setting
- Psychosocial/mental health
- Private center
- Private sessions
- Other (please specify) ___________________________________

1. How many occupational therapists work in your clinical setting?

- One therapist (including yourself)
- Two therapists (including yourself)
- More than two therapists (including yoursel

1. How many hours per week do you work as an OT?

- 0-4 hours
- 5–9 hours
- 10–14 hours
- 15–19 hours
- 20–24 hours
- 25–29 hours
- 30–34 hours
- 35–39 hours
- >39 hours

1. Are you a part of any association?

If yes, choose "other" and write its name

- No
- Other:

1. What university you graduated from? *

- The University of Jordan
- The Hashemite University
- Jordan University of science and technology
- Other:

1. In which city your job is located?

__________________________________

**Section 2:** Sources of information for clinical decision-making.

I would like also to know more about the evidence sources that you use for making clinical decisions. For the following questions, please indicate how often you use the following information sources in your clinical practice.

| I use the following …. | Always (100%) | Often (75%) | Sometimes (50%) | Rarely (25%) | Never | I have no access to this source |
| --- | --- | --- | --- | --- | --- | --- |
| - Information obtained from clinical experience |  |  |  |  |  |  |
| - Information obtained from conferences and workshops |  |  |  |  |  |  |
| - Information obtained from reviewing research articles |  |  |  |  |  |  |
| - Information obtained from textbooks |  |  |  |  |  |  |
| - Information obtained from occupational therapy colleagues |  |  |  |  |  |  |
| - Information obtained from non- occupational therapy colleagues |  |  |  |  |  |  |
| - Information obtained from web resources |  |  |  |  |  |  |
| Other: ________________ |  |  |  |  |  |  |

**Section 3:** Attitudes toward the importance of EBP into clinical practice.

In this section, I would like to learn more about your feelings about utilizing EBP in the clinical setting. For the following items, please select the choice that best describes your beliefs about evidence-based practice (EBP).

| I believe that ….. | Strongly agree | Agree | Neither agree nor disagree | Disagree | Strongly disagree |
| --- | --- | --- | --- | --- | --- |
| 1. Evidence-based practice can play a positive role in clinical practice. |  |  |  |  |  |
| 1. Evidence-based practice helps with clinical decision-making. |  |  |  |  |  |
| 1. Evidence-based practice improves patient outcomes. |  |  |  |  |  |
| 1. I would like to use evidence in clinical practice. |  |  |  |  |  |
| 1. Academics are more likely to use evidence-based practice than clinicians. |  |  |  |  |  |
| 1. Evidence-based practice is difficult to use in clinical practice. |  |  |  |  |  |
| 1. The profession should emphasize research in its education. |  |  |  |  |  |
| 1. Evidence-based practice devalues practitioners’ clinical experience. |  |  |  |  |  |
| 1. Evidence-based practice removes creativity from practice. |  |  |  |  |  |
| 1. Research is essential for demonstrating the efficacy of interventions. |  |  |  |  |  |
| 1. Research and clinical experience are equally important. |  |  |  |  |  |
| 1. Evidence-based practice is client-centered. |  |  |  |  |  |
| 1. Research is essential to the occupational therapy profession. |  |  |  |  |  |
| 1. Studying research is a waste of time. |  |  |  |  |  |
| 1. I do not feel the value of evidence-based practice. |  |  |  |  |  |

(Questions based off Crane, Pelz, and Horsley 1997, Dӧpp et al., 2012; Valdes &Heyde, 2012)

**Section 4:** Barriers influencing the use of EBP

For this section, I would like to identify which barriers influence the use of EBP in clinical setting. Please select the choice that best describes your opinion about barriers to utilizing EBP.

|  | Strongly agree | Agree | Neither agree nor disagree | Disagree | Strongly disagree |
| --- | --- | --- | --- | --- | --- |
| - 1. Occupational therapists do not have time to read research. |  |  |  |  |  |
| - 3. There is limited access to research articles. |  |  |  |  |  |
| - 4. Occupational therapists lack skills for locating the best research evidence. |  |  |  |  |  |
| - 5. Occupational therapists lack skills for evaluating and understanding results of research. |  |  |  |  |  |
| - 7. Occupational therapists do not have enough authority to implement new ideas from the literature into practice. |  |  |  |  |  |
| 1. Reimbursement is based on quantity of cases instead of quality of intervention. |  |  |  |  |  |
| 9. There is insufficient time to implement new ideas obtained from research. |  |  |  |  |  |
| - 10. Occupational therapists find it difficult to change their established patterns of practice. |  |  |  |  |  |
| - 11. Tools and equipment in clinical settings are inadequate for the implementation of EBP. |  |  |  |  |  |
| - 12. There is a lack of incentive for using evidence-based practice. |  |  |  |  |  |

(Questions based off Crane, Pelz, and Horsley 1997, Dӧpp et al., 2012; Valdes &Heyde, 2012)

- Which of the listed above items do you feel are the *three greatest barriers* to OTs’ use of evidence based practice?

1. Greatest Barrier Item #:
2. Second Greatest Barrier Item #:
3. Third Greatest Barrier Item #:

**Section 5:** In this section, I would like to know more about your use of utilizing EBP in clinical decision-making.

Please indicate the approximate percentage of clinical decision-making time that involves evidence-based practice

- 0% - 25%
- 26% - 50%
- 51% - 75%
- 76% - 100%
- What resources would help you to incorporate evidence-based practice more frequently in the occupational therapy treatment process?

Thank you for taking the time to participate in the survey, and helping to contribute valuable information to this project.

Your responses are greatly appreciated!
